# Children’s Exposure to Radon in Nursery and Primary Schools

**DOI:** 10.3390/ijerph13040386

**Published:** 2016-03-30

**Authors:** Pedro T. B. S. Branco, Rafael A. O. Nunes, Maria C. M. Alvim-Ferraz, Fernando G. Martins, Sofia I. V. Sousa

**Affiliations:** LEPABE—Laboratory for Process Engineering, Environment, Biotechnology and Energy, Faculty of Engineering, University of Porto, Rua Dr. Roberto Frias, 4200-465 Porto, Portugal; p.branco@fe.up.pt (P.T.B.S.B.); raonunes@fe.up.pt (R.A.O.N.); aferraz@fe.up.pt (M.C.M.A.-F.); fgm@fe.up.pt (F.G.M.)

**Keywords:** radon, exposure, children, nursery, primary school

## Abstract

The literature proves an evident association between indoor radon exposure and lung cancer, even at low doses. This study brings a new approach to the study of children’s exposure to radon by aiming to evaluate exposure to indoor radon concentrations in nursery and primary schools from two districts in Portugal (Porto and Bragança), considering different influencing factors (occupation patterns, classroom floor level, year of the buildings’ construction and soil composition of the building site), as well as the comparison with IAQ standard values for health protection. Fifteen nursery and primary schools in the Porto and Bragança districts were considered: five nursery schools for infants and twelve for pre-schoolers (seven different buildings), as well as eight primary schools. Radon measurements were performed continuously. The measured concentrations depended on the building occupation, classroom floor level and year of the buildings’ construction. Although they were in general within the Portuguese legislation for IAQ, exceedances to international standards were found. These results point out the need of assessing indoor radon concentrations not only in primary schools, but also in nursery schools, never performed in Portugal before this study. It is important to extend the study to other microenvironments like homes, and in time to estimate the annual effective dose and to assess lifetime health risks.

## 1. Introduction

Radon is by far the most important source of ionizing radiation among those of natural origin. It is a natural radioactive gas with origin in the decay of uranium that is found in soil and rocks. It is a colourless, odourless and tasteless gas that travels through the soil and enters buildings through cracks in the foundations. The radioactive particles (decay products) from radon decay during breathing can be retained in the lungs, continuously releasing ionizing radiation that can harm human health [1].

Radon levels in dwellings are usually subject to a typical diurnal variation with higher concentrations during the night and dawn, and are also subject to a typical seasonal variation with the highest concentrations during the heating season (October to April) [1]. Annual random variations are also usual, and they can be related to several factors, such as weather patterns and occupants’ behavior [2]. The worldwide average indoor radon concentration has been estimated at 39 Bq·m^−3^ [1].

Epidemiological studies around the world have provided evidence of an association between exposure to radon inside buildings and lung cancer, even at the relatively low levels commonly found in residential buildings [3]. Radon is considered by the United States Environmental Protection Agency (USEPA) and the World Health Organization (WHO) as the main cause of lung cancer among non-smokers and the second cause of lung cancer in the general population (smoking being first) and there is no known threshold below which exposure to radon does not present risk [1,4].

Therefore, the International Committee for Radiological Protection (ICRP) has emphasized the importance of monitoring and controlling radon concentrations in dwellings and work places [5]. For the latter, kindergartens and primary schools present a special case, being the workplace for the childcare workers and a living environment for children [6]. In fact, children spend more time in school environments (including nursery schools) than in any other indoor environment besides home [7]. Children are considerably more sensitive to the carcinogenic effects of ionizing radiation than adults [8], even at low doses, as well as those from natural radiation [9]. However, until now there is no epidemiological evidence that children are at greater risk than adults from radon exposure [10]. Despite this, a special interest has been observed in indoor radon measurements in nursery and primary schools with studies being performed in many parts of the world [6]. 

Considering the latter and as far as the authors’ knowledge goes, in the last five years 19 studies regarding radon in indoor air of nursery and primary schools can be found. The majority of them were only focused on primary schools [11,12,13,14,15,16,17,18,19,20,21], while others also considered nursery schools [10,22,23,24,25,26] and some others only considered nursery schools [27,28]. The extent of the studies varied considerably from study to study. There are studies (surveys) that included a considerable number of nursery and primary schools of a particular country or region [14,16,27] and others studied only a limited number of buildings [25,26]. In the latter cases, a representative number of buildings was selected taking into account selection criteria that varied from case to case, like a comparison between two different cities [10] or a comparison between urban and rural contexts [18]. In most of the above referred recent studies, indoor radon concentrations were measured over a fixed time period using passive solid-state nuclear track detectors (CR-39 track detectors) and electret ionization chambers (EIC), although some studies used electronic devices such as electronic integrating devices (EID) and continuous radon monitors (CRM). In fact, CRM allows understanding differences in radon concentrations between occupation and non-occupation periods as well as the baseline room scenario. In order to investigate the influence of different factors on indoor radon concentrations the majority of the studies considered measurements in different locations and different floor levels inside the buildings. Furthermore, some authors considered other factors such as type of use, building age, building materials, building improvements and different geographic contexts (rural *vs.* urban). 

Considering the latest scientific data, the WHO [1] proposes an annual reference average level of 100 Bq·m^−3^ to minimize health risks due to exposure to radon inside buildings. However, if this cannot be achieved under the specific conditions of the country, the chosen reference level should not exceed 300 Bq·m^−3^. USEPA [4] recommends radon concentrations below 4 pCI·L^−1^ (148 Bq·m^−3^). The European Union (EU) recommends annual average radon concentrations below 300 Bq·m^−3^ in dwellings and mixed-use buildings, like primary and nursery schools [29]. In Portugal, the existing legislation sets a limit of 400 Bq·m^−3^ (annual mean value) and mandatory measurements in granitic areas, namely in the districts of Braga, Porto, Vila Real, Guarda, Viseu and Castelo Branco [30].

Thus, as part of the INAIRCHILD project [31], and following the previously preliminary study with the same sampling methodology but covering only three nursery schools and one primary school [23], this study aimed to evaluate indoor radon concentrations to which children were exposed in nursery and primary schools from two different districts in Portugal (Porto and Bragança), considering different influence factors (occupation patterns, classroom floor levels, year of buildings’ construction and soil composition of the building site), as well as the comparison with IAQ standard values for health protection. As far as the authors know this study presents a new approach to the study of children’s radon exposure by using continuous measurement of indoor radon concentrations inside nursery and primary schools (some of these buildings were already used in a preliminary study) [23], in different districts, and associated with the assessment of the influence of several factors. 

## 2. Materials and Methods

### 2.1. Site Description

This study was carried out in fifteen different school buildings in Northern Portugal, of which seven were located in the Porto district—an urban radon-prone area, and the remaining eight were located in Bragança district—a rural area that is not considered mandatory for indoor radon measurements according to Portuguese IAQ legislation [30]. Studied were a total of five nursery schools for infants (children aged under 3) and twelve for pre-schoolers (3–5 years old), as well as eight primary schools (children aged 6–10 years old). One or more classrooms in each nursery and primary school were considered for this study. Lunch rooms were always sampled. From those, three nursery schools and one primary school from the Bragança district were the same as in a previous study [23]. A prior inspection (through direct observations and interviews with the staff) was performed to capture relevant information on timetables, activities and occupation, ventilation and other building characteristics that could be relevant to analyze the results obtained in this study. Table 1 summarizes the characterization of the studied microenvironments, namely the distribution of the buildings per year of construction and regarding soil composition, namely the predominant types of rocks in the soil according to the Geological Map of Portugal [32], as well as the number of classrooms per floor level in the building, for both districts and according to the occupation in each one. Regarding occupation, three different subsets were considered according to the age of the children (occupants): infants, pre-schoolers and school children.

The majority of the buildings analysed were built before 2006, *i.e.*, before the implementation of the first Portuguese legislation on IAQ (transposition of the European Directive including a radon reference level), and some of them were even centenary. Although renovations had recently been performed in some nursery and primary schools, the analyses were performed considering the year of construction of the building (the only exception was a building in the Porto district used as both a nursery and primary school initially built before 2006, but totally rebuilt recently).

The majority of the studied classrooms were located on the ground floor of the buildings, and natural ventilation was predominant. In fact, only four of the studied classrooms had predominant forced ventilatation (mechanical ventilation): two in the Porto district and another two in the Bragança district, all used for infants. 

The majority of the buildings studied in the Porto district were placed on soils where magmatic rocks are predominant, but in the Bragança district they were mainly located on soils with metamorphites as predominant rocks. There was only one building placed on a predominantly sedimentary soil—a nursery school used both for infants and pre-schoolers in the Porto district. To the best of the authors’ knowledge, none of the studied buildings were built on slabs nor did they have a basement-like area.

### 2.2. Sampling

Radon measurements were made continuously (logging hourly means) using a Radim 5B radon monitor (SMM, Prague, Czech Republic), which measures the α-activity of radon decay products (^218^Po and ^214^Po) collected from the detection chamber on the surface of a semiconductor detector by an electric field. 

This radon monitor was calibrated by the manufacturer by placing it in a barrel (controlled atmosphere) next to a reference instrument (Radim 3, verified in the Metrological Institute of Czech Republic), and recording measurements with both instruments simultaneously during about 24 h. The results of both instruments were compared and CAL factor of the calibrated equipment was modified (3.6%) to get the same result as in the reference instrument. Calibration precision was about 5%. The error of the equipment is 5% for concentrations above 80 Bq·m^−3^, and 20% for concentrations below that. 

The equipment was placed as close to the centre of the room as possible, far from windows, doors and room’s corners, approximately at the same height of children’s breath (1.5 ± 0.5 m). Depending on secured permissions, and due to financial constraints, short-term samplings were performed from 2 to 9 consecutive days in each room (not simultaneously) in nursery schools, and for at least 24 h in primary schools, and, in some circumstances, both on weekdays and weekends.

### 2.3. Data Analysis

Descriptive statistics were initially determined, namely mean, median, minimum, maximum and standard deviation. Histograms were drawn to take a look at data and normality of the distributions was assessed through both Kolmogorov-Smirnov, Shapiro-Wilk and Anderson-Darling normality tests. Distributions were assessed for log-normality using the same normality tests applied after a logarithmic data transformation. 

The non-parametric Wilcoxon Rank Sum Test (also called Mann-Whitney *U* test) was used to test the significance of the differences between two samples, and the non-parametric Kruskal-Wallis test was used when comparing the significance of the differences between three samples.

Descriptive statistics were calculated using MS Excel^®^ (Microsoft Corporation, Redmond, WA, USA), and other statistical analyses were determined using R software, version 3.1.2 (R Foundation for Statistical Computing, 2014).

Although the mean radon indoor concentrations measured were preliminary, as they result from short-term sampling (screening), they were compared with IAQ standards and guidelines for health protection (annual) attempting to preliminarily evaluate exceedances. Comparisons were performed considering international references, namely: (a) WHO reference values of 100 Bq·m^−3^ and 300 Bq·m^−3^ [1]; (b) USEPA reference value of 4 pCI·L^−1^ (148 Bq·m^−3^) [4]; (c) EU reference value of 300 Bq·m^−3^ [29]; and (d) Portuguese reference value of 400 Bq·m^−3^ [30]. 

## 3. Results and Discussion

In the studied nursery and primary schools in the Porto district, indoor radon concentrations varied from 0 to 459 Bq·m^−3^ (*N* = 2429), with an average (standard deviation) of 62 ± 86 Bq·m^−3^. In the studied nursery and primary schools in the Bragança district, concentrations varied from 0 to 888 Bq·m^−3^ (*N* = 1342), with an average (standard deviation) of 193 ± 174 Bq·m^−3^. 

**Figure 1:**
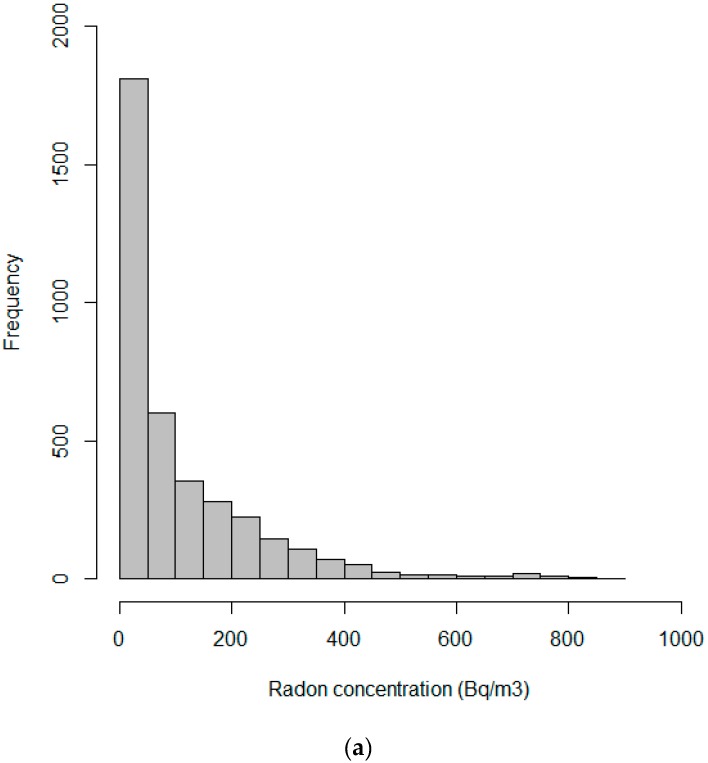

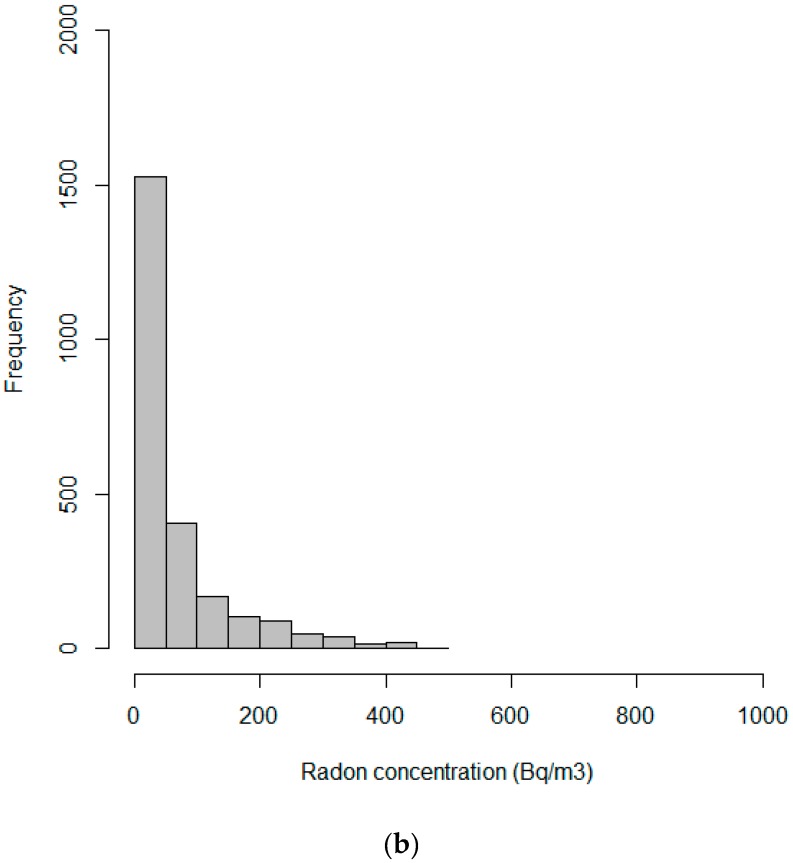

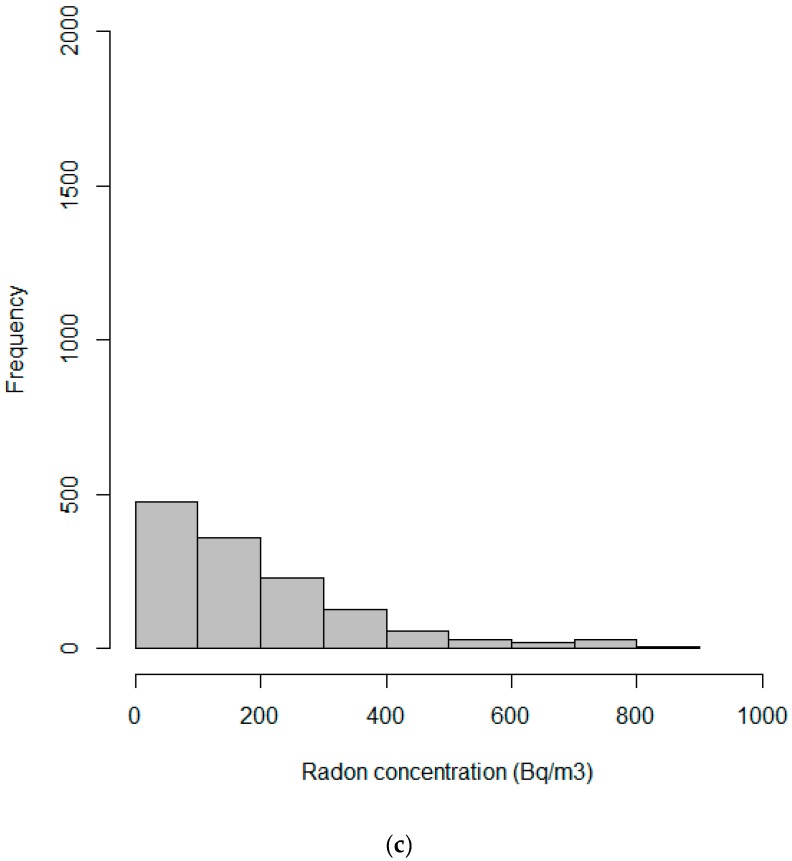
Frequency distribution of the hourly indoor radon concentrations measured in (**a**) all the studied buildings; (**b**) Porto district and (**c**) Bragança district.

Indoor radon concentrations were found to be significantly higher in the nursery and primary schools in Bragança than in the Porto district (*p* < 0.05). Figure 1 represents the frequency distribution of the hourly indoor radon concentrations obtained in each studied classroom of: (a) all the studied buildings; (b) the buildings in the Porto district; and (c) the buildings in the Bragança district. Low concentrations were the most common, and the highest indoor concentrations were found in the studied microenvironments in Bragança district. The Figure 1 data distributions look like log-normal ones, nevertheless, the results from Kolmogorov-Smirnov, Shapiro-Wilk and Anderson-Darling normality tests (after a logarithmic data transformation) for the data from nursery and primary schools in the Porto and Bragança districts showed that data did not follow a normal distribution (*p* < 0.05).

**Figure 2:**
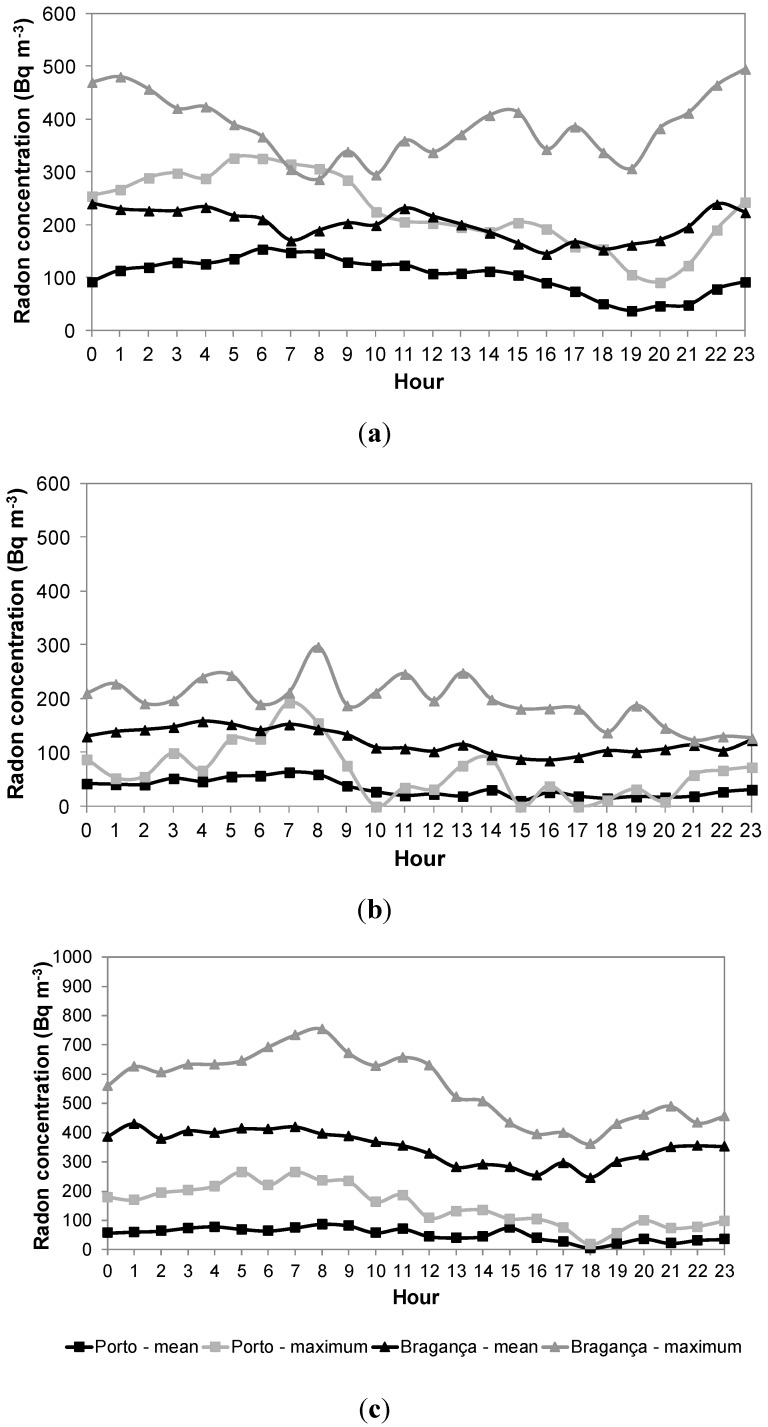
Daily mean and maximum scenarios of indoor radon concentrations in ground floor classrooms in Porto and Bragança districts for (**a**) infants; (**b**) pre-schoolers; and (**c**) school children.

Continuous sampling allowed assuming an average scenario, *i.e.*, hourly mean concentrations between two or more consecutive days were calculated, allowing the representation of the mean daily profile. As an example, Figure 2 shows two different scenarios for daily profiles considering ground floor classrooms for: (a) infants; (b) pre-schoolers; and (c) school children: (i) the mean concentrations (daily mean scenario, in black); and (ii) the classroom with the highest mean concentrations found (daily maximum scenario, in grey).

From Figure 2 it was possible to observe a similar pattern in the daily mean profiles of indoor radon concentrations in all the studied microenvironments: an increase of indoor concentration at the end of the day resulting in higher concentrations during night and dawn, followed by a decrease along the day. 

**Figure 3:**
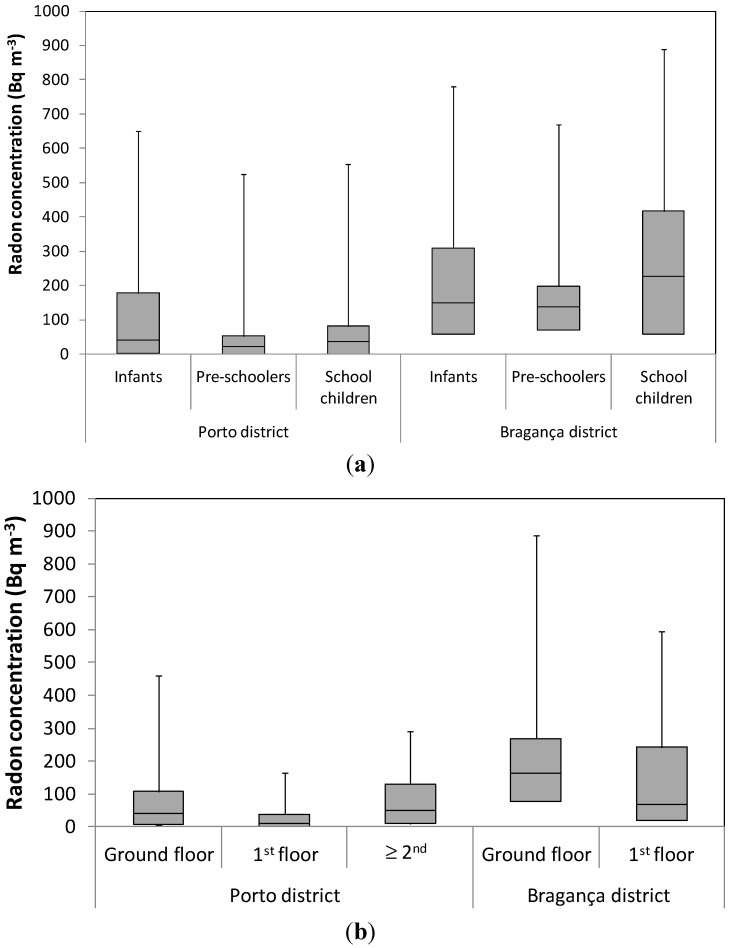

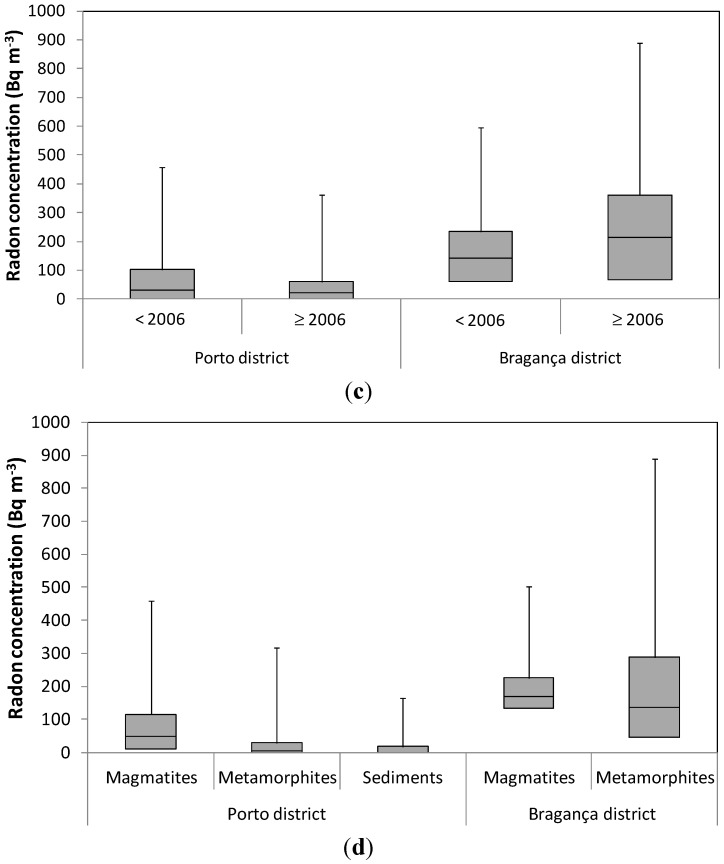
Distribution of radon concentrations in the studied microenvironments in both Porto and Bragança districts per (**a**) occupation; (**b**) classroom floor; (**c**) year of buildings’ construction; and (**d**) soil composition of the building site.

Occupation patterns seemed to be responsible for those profiles, with higher concentrations at night caused by the absence of air renovation (accumulation), and with lower concentrations along the day due to a higher air turnover (through natural or mechanical ventilation). These patterns are in accordance with the typical daily patterns found in the literature for dwellings [1]. Indoor radon concentrations were grouped according to some of the main influence factors, namely occupation, classroom floor level, year of buildings’ construction and soil composition of the building site. Figure 3 presents the distribution of radon concentrations in the studied microenvironments in both the Porto and Bragança districts per subsets, namely: (a) occupation; (b) classroom floor level; (c) year of buildings’ construction; and (d) soil composition of the building site. 

While indoor radon concentrations in the studied microenvironments in the Porto district were found to be higher in the classrooms occupied by infants and lower in the classrooms occupied by pre-schoolers, in the studied microenvironments in Bragança they were found to be higher in the primary schools’ classrooms and lower in the classrooms for pre-schoolers. Results indicated statistical significant differences between indoor radon concentrations in the classrooms depending on the occupation both in the classrooms studied in the Porto and Bragança districts (*p* < 0.05). This seemed to be caused by the different activity patterns in classrooms which are highly dependent of the childrens’ development stage: infancy, pre-school age or school age. Therefore, it is expected to find different results when assessing indoor radon concentrations in classrooms for infants, pre-schoolers or school children and it should be considered when assessing indoor radon concentrations in scholar buildings. In fact, in the studied nursery schools in the Porto district, infants are expected to be exposed to higher radon concentrations than older children (pre-schoolers and school children), although in those in the Bragança district the results indicated that the highest concentrations are expected to be inhaled by school children, followed by infants and pre-schoolers.

All differences found between measurements in the different floor levels were found to be statistically significant (*p* < 0.05). As expected, higher indoor radon concentrations were found in the ground floor classrooms in both districts, which is the closest floor to the soil—the main source of radon in indoor air. It is important to take this into account when assigning rooms usage in school buildings. Nevertheless, indoor radon concentrations in the studied microenvironments in the Porto district were found to be higher in the 2nd and higher floor classrooms than in the 1st floor classrooms, which might be associated with the limited data in this particular case—a one-off case where higher values occurred in a 2nd or higher classroom due to radon flow through cracks in the building. 

Statistically significant differences (*p* < 0.05) were always found between indoor radon concentrations in the studied buildings built before 2006 and those built after 2006, both in the Porto and Bragança districts. In the Porto district, the older buildings studied (built before the promulgation of the first IAQ Portuguese legislation) registered higher indoor radon concentrations than the newer ones (built in 2006 and afterwards). This might indicate that the introduction of the IAQ Portuguese legislation (introduction of a limit value for radon in indoor air) had an important role in reducing indoor radon concentrations inside buildings. On the other hand, in the Bragança district the newer buildings studied (built in 2006 and after) registered the highest indoor radon concentrations, which might indicate that, despite the introduction of the IAQ Portuguese legislation, as indoor radon measurements were not mandatory in Bragança district nothing seemed to have been done to prevent high concentrations inside the buildings in that district.

The nursery and primary schools from Porto district built upon soils where magmatites were predominant registered higher indoor radon concentrations than those where metamorphites were predominant, which in turn registered higher indoor radon concentrations than those where sediments prevailed. These results are in agreement with what was initially expected [33]. However, in the Bragança district, the studied nursery and primary schools built upon soils where metamorphites predominate registered higher indoor radon concentrations than those where magmatites prevailed. All the differences were found to be statistically significant (*p* < 0.05). Since the results were the opposite in the studied buildings in the two districts, without a deeper analysis it is not possible to understand the real influence that soil composition of the building site has on the indoor radon concentrations found. Thus, it could be dangerous to limit indoor radon assessment to buildings constructed in a specific type of soil. The influence of other factors in the radon indoor concentrations, like building materials [34], could be also important, but it was not performed in this study because data was not available.

Although the mean radon indoor concentrations measured were preliminary, as they result from short-term sampling (screening), attempting to preliminarily evaluate exceedances, indoor radon mean concentrations during occupation periods in the studied microenvironments were compared with the reference values for IAQ and health protection referred in Section 2.3 and exceedances were calculated (*i.e.*, the number of classrooms where the mean indoor concentration during occupation period was above the reference value). 

In the Porto district, the majority of the classrooms assessed (25/30) did not exceed the reference values considered, while in the Bragança district only some of the studied classrooms did not show exceedances (6/17). In fact, indoor radon concentrations found in the studied nursery and primary schools in the Porto district never exceeded the reference radon indoor concentration from the Portuguese national legislation on IAQ (the least restrictive of all the reference values here considered), although in the Bragança district two classrooms exceeded it—one from a nursery and the other from a primary school. Classrooms for pre-school children in Porto never exceeded any of the reference values, and only two classrooms of primary schools exceeded the WHO reference value of 100 Bq·m^−3^ (the most restrictive). In the case of classrooms for infants, only three exceeded both WHO and USEPA reference values (100 and 150 Bq·m^−3^, respectively). In the Bragança district, three of the classrooms for infants exceeded the WHO reference value of 100 Bq·m^−3^, two of them also exceeded USEPA reference value and only one of them exceeded both the WHO and EU reference values of 300 Bq·m^−3^ as well as the Portuguese legislation. Four of the studied classrooms for pre-schoolers exceeded the WHO reference value of 100 Bq·m^−3^ and two of them also exceeded the USEPA reference value. Four of the primary schools’ classrooms analysed exceeded the WHO guideline of 100 Bq·m^−3^, three of them also exceeded the USEPA reference value, and one of them also exceeded both the WHO and EU reference of 300 Bq·m^−3^, as well as the Portuguese legislation.

Although indoor radon concentrations were in general within the Portuguese legislation for IAQ, a considerable number of exceedances to the international reference values for IAQ and health protection were found, which is a concerning situation as there is no known threshold below which radon inhalation exposure does not present any risk to human health [1]. 

Eighteen studies published in the last 5 years were found in the literature regarding radon levels in the indoor air of school microenvironments. Table 2 summarizes the main characteristics of those studies and the mean radon indoor concentrations that they reported.

Indoor mean radon concentrations reported in recent literature for nursery schools from Italy [24], Bulgaria [26], Slovenia [25] and Czech Republic [27] were usually higher than those found in the present study, in both school microenvironments in the Porto and Bragança districts, which in turn were higher than those found in nursery schools from Saudi Arabia [22] and Poland [10]. 

On the other hand, indoor radon concentrations found in schools from the Porto district were lower than the majority of those found in recent literature, except when comparing with the results from Turkish [15], Canadian [17] and Pakistani schools [18,19]. Indoor radon concentrations found in schools from the Bragança district were higher than the majority of the ones reported in the literature, except for those in Bulgaria [26] and Slovenia [25], which enhance the concerns about the results here found for the Bragança district.

Despite the limitations of this new preliminary approach, this study shows that the results found were quite concerning from the children’s health point of view, especially in the Bragança district, because radon is a carcinogenic compound and its inhalation has been associated with lifetime lung cancer risk. It also points out the need of assessing indoor radon concentrations not only in primary schools, but also in nursery schools, since children are expected to be exposed to relevant concentrations from infancy in those microenvironments. These preliminary data will be useful for the future survey of the long-term radon concentrations measurements.

## 4. Conclusions

Continuous active sampling allowed understanding the daily profile of indoor radon concentrations in both nursery and primary schools from the Porto and Bragança districts. The results showed higher concentrations during night and dawn caused by the absence of air renewal, and lower along the day due to a higher air renovation. These patterns were found to be in accordance with the typical daily patterns already reported in the literature for other types of dwellings.

Classroom occupation (determined by children’s age, activities and number of occupants) influenced radon concentrations inside classrooms. Thus, different results can be expected when assessing indoor radon concentrations in scholar microenvironments occupied by infants, pre-schoolers or school children. Another significant factor was floor level, with higher concentrations registered in the lower floors (the closest to the soil which is the main source of indoor radon). Consequently, floor level should be considered when assigning rooms usage in school buildings. The year of a building’s construction, namely before or after 2006 (introduction of the IAQ Portuguese legislation), seemed to have had an important role in reducing indoor radon concentrations inside the studied buildings in the Porto district. Limiting indoor radon assessment based upon buildings constructed in a specific type of soil will be difficult, because the results did not allow understanding the real influence that soil composition of the building site has in the indoor radon concentrations and in the radon children’s exposure in the nursery and primary schools. These results point out the need of assessing indoor radon concentrations not only in primary schools, but also in nursery schools, since children are expected to be exposed to relevant concentrations from infancy in those microenvironments. Radon is a carcinogenic compound, its inhalation has been associated with lifetime lung cancer risk and in fact, there is no known threshold below which radon inhalation exposure does not present risk to human health, so the results were quite concerning, especially in the studied nursery and primary schools from the Bragança district. 

Short-time measurements as well as a limited number of classrooms and buildings studied constituted a study limitation, thus this study is considered a preliminary assessment. Nevertheless, these preliminary data will be useful for the future survey of the long-term radon concentrations measurements. In the future it could be important to use the same approach in a national indoor radon survey in nursery and primary schools, as recommended by the International Commission on Radiological Protection [35] and by the European Commission [29], studying more classrooms and buildings and using long-term measurements which will allow estimates of the annual effective inhaled dose. It could also be important to study the influence of other factors, like building materials. To extend the study to other indoor microenvironments, like homes, will allow to determinate daily children’s exposure to radon in indoor air with more accuracy and it will also allow to assess the lifetime health risks and to estimate the related lung cancer deaths [36].

## Figures and Tables

**Table 1:** Characterization of the studied buildings according to the year of construction, the type of predominant rocks in the soil (main soil composition), and the classrooms’ floor level in the building.

Occupation	Number of Buildings						Number of Classrooms			
	Total	Year of Construction		Predominant Rock Type in the Soil			Total	Classrooms’ Floor Level		
		Before 2006	2006 or after	Magmatites	Metamorphites	Sediments		Ground Floor	1st Floor	2nd and Higher Floors
Porto district	7 ^a^	3 ^a^	4 ^a^	5 ^a^	1 ^a^	1 ^a^	30	17	10	3
Infants (<3 years old)	3	1	2	2	0	1	6	4	2	0
Pre-schoolers (4–5 years old)	7	3	4	5	1	1	13	7	5	1
School children (6–10 years old)	5	3	2	4	1	0	11	6	3	2
Bragança district	8 ^a^	2 ^a^	6 ^a^	3 ^a^	5 ^a^	0 ^a^	17	14	3	0
Infants (<3 years old)	2	1	1	0	2	0	4	4	0	0
Pre-schoolers (4–5 years old)	5	2	3	2	3	0	7	7	0	0
School children (6–10 years old)	3	1	2	1	2	0	6	3	3	0

^a^ There are situations in which both classrooms for infants and pre-schoolers, and for pre-schoolers and school children were in the same building.

**Table 2:** Summary of the main results of most recent studies (last 5 years) regarding radon in indoor air of school microenvironments.

Location	Type of Schools	Concentration (Bq·m^−3^)	References
Bulgaria (Kremikovtsi)	Nursery and primary schools	339 (short term)	Vuchkov *et al.* [26]
		694 (long term)	
Saudi Arabia (Zulfi)	Primary schools	80.0	Al-Ghamdi *et al.* [22]
	Nursery schools	80.1	
Greece	Primary schools	149	Clouvas *et al.* [14]
Italy (South-East)	Primary schools	218	Trevisi *et al.* [24]
	Nursery schools	246	
Turkey (Batman)	Primary schools	46	Damla and Aldemir [15]
Poland (Kalisz) Poland (Ostrów Wielkopolski)	Nursery and primary schools	46.0 48.9	Bem *et al.* [10]
Serbia (Southern)	Primary schools	119	Bochicchio *et al.* [11]
	Primary schools	118	Zunic *et al.* [21]
Canada (Province of Quebec)	Primary schools	56	Poulin *et al.* [17]
Slovenia	Nursery schools	145 to 794	Vaupotic *et al.* [25]
	Primary schools	70 to 770	
Bulgaria (Sofia)	Nursery schools	132	Ivanova *et al.* [28]
Republic of Macedonia	Primary schools	88	Stojanovska *et al.* [20]
Czech Republic	Nursery schools	204 (reconstruction) 149 (non-reconstruction)	Fojtikova and Navratilova Rovenska [27]
Korea (Some provinces)	Primary schools	23 to 1414	Chang *et al.* [13]
Korea (National survey)	Primary schools	98.4	Kim *et al.* [16]
Pakistan (Punjab)	Primary schools	52	Rahman *et al.* [19]
Pakistan (Urban area)	Primary schools	39	Rahman *et al.* [18]
Pakistan (Rural area)	Primary schools	47	
Romania (3 counties)	Primary schools	215	Burghele and Cosma [12]
Portugal (Porto district)	Nursery schools (infants)	101	This study
	Nursery schools (pre-schoolers)	37	
	Primary schools	57	
Portugal (Bragança district)	Nursery schools (infants)	189	
	Nursery schools (pre-schoolers)	138	
	Primary schools	275	
